# A Tent Marine Predators Algorithm with Estimation Distribution Algorithm and Gaussian Random Walk for Continuous Optimization Problems

**DOI:** 10.1155/2021/7695596

**Published:** 2021-12-28

**Authors:** Chang-Jian Sun, Fang Gao

**Affiliations:** ^1^College of Electronic Science and Engineering, Jilin University, Changchun 130012, China; ^2^College of Computer Science and Technology, Jilin University, Changchun 130012, China; ^3^Chang Guang Satellite Technology Co., Ltd, Changchun 130000, China

## Abstract

The marine predators algorithm (MPA) is a novel population-based optimization method that has been widely used in real-world optimization applications. However, MPA can easily fall into a local optimum because of the lack of population diversity in the late stage of optimization. To overcome this shortcoming, this paper proposes an MPA variant with a hybrid estimation distribution algorithm (EDA) and a Gaussian random walk strategy, namely, HEGMPA. The initial population is constructed using cubic mapping to enhance the diversity of individuals in the population. Then, EDA is adapted into MPA to modify the evolutionary direction using the population distribution information, thus improving the convergence performance of the algorithm. In addition, a Gaussian random walk strategy with medium solution is used to help the algorithm get rid of stagnation. The proposed algorithm is verified by simulation using the CEC2014 test suite. Simulation results show that the performance of HEGMPA is more competitive than other comparative algorithms, with significant improvements in terms of convergence accuracy and convergence speed.

## 1. Introduction

Solving optimization problems in engineering and scientific research is a common problem. An optimization problem is the process of finding the best value of a decision variable that satisfies the maximum or minimum objective value without violating the constraints. Traditional gradient-based deterministic algorithms show difficulty in solving practical problems [1]. With the development of science and technology today, the optimization problems we encounter are becoming more and more complex. These real-world optimization problems often involve many decision variables, complex nonlinear constraints and nonconvexity, dynamic objective functions, and expensive computational costs [2, 3]. Although these algorithms achieve faster processing speeds, they can easily fall into local optima. In addition, the performance of the algorithms depends heavily on the characteristics of the problem and the initial parameter values. However, metaheuristic algorithms, which do not depend on the characteristics of the problem, are simple in structure, flexible, and do not rely on gradient information and have therefore received widespread attention and flourished among scholars. As a result, it is widely used to solve various optimization problems, such as task planning [4, 5], feature selection [6, 7], parameter optimization [8, 9], and image segmentation [10, 11].

Over the past decades, many metaheuristic algorithms have been proposed. These algorithms can be divided into three categories: evolution-based algorithms, physics-based algorithms, and swarm-based algorithms. Evolutionary-based algorithms are a class of algorithms that simulate the laws of evolution in nature. Genetic algorithm (GA) [12] is a widely used evolution-based algorithm proposed by Holland. It updates populations by simulating the natural law of superiority and inferiority. With the popularity of GA and GA variants, more and more evolutionary-based algorithms have been proposed, including differential evolution (DE) [13], genetic programming (GP) [14], and evolutionary strategy (ES) [15]. In addition to these evolutionary algorithms, new evolutionary-based algorithms have recently been proposed, such as the artificial algae algorithm (AAA) [16] and monkey king evolutionary (MKE) [17]. Physics-based algorithms simulate the laws of physics in nature or in the universe. Inspired by the phenomenon of annealing in metallurgy, simulated annealing (SA) [18] is one of the best-known physics-based algorithms. Other physics-based algorithms have been proposed, including the gravitational search algorithm (GSA) [19], nuclear reaction optimization (NRO) [20], water cycle algorithm (WCA) [21], and sine cosine algorithm (SCA) [22]. Population-based algorithms simulate the social behaviour of species such as self-organisation and division of labour. Particle swarm optimization (PSO) [23] and ant colony optimization (ACO) [24] are two classical swarm-based algorithms. Inspired by these two algorithms, an increasing number of scholars have studied this topic and proposed different swarm-based algorithms such as grey wolf optimizer (GWO) [25], whale optimization algorithm (WOA) [26], sparrow search algorithm (SSA) [27], firefly algorithm (FA) [28], artificial bee colony algorithm (ABC) [29], and tuna swarm optimization (TSO) [30].

The marine predators algorithm (MPA) is a novel population-based natural heuristic optimization algorithm proposed by Faramarzi et al. [31], which is mainly inspired by the different foraging strategies of marine predators and the optimal encounter rate strategies between predators and prey. Simulation results in the literature [31] show that MPA has better performance compared to GA, PSO, GSA, CS, SSA, and CMA-ES and thus has been widely used to tackle many practical engineering problems such as photovoltaics [32, 33], power systems [34], image classification [35], and task scheduling [36].

Although MPA has been applied in several fields, there is less analysis and improvement on the shortcomings of MPA. MPA mainly searches near the optimal individuals when performing population position update, without using the effective information of more individuals. The insufficient diversity leads the MPA into a local optimum. The FADs process is designed to enhance the diversity of the population, but it does not determine the superiority of the offspring and the parent, which is not conducive to the optimization and convergence of the algorithm to a certain extent.

Currently, there are three main approaches to improve the performance of natural heuristic optimization algorithms. The first is parameter tuning. Tang et al. [37] used chaos mapping to optimise key parameters of sparrow search algorithm. Ewees and Elaziz [38] investigated the effect of different chaotic mapping tools on the parameter settings of the algorithm. The second approach is to design different search operators. Zhang et al. [39] used a triangular variational strategy and a logarithmic spiral strategy to improve the development and exploration performance of the algorithm. Nor et al. [40] proposed an adaptive switching particle swarm algorithm based on a hybrid update sequence. The third approach is to introduce other techniques. The fractional order is an effective tool that has been used in other areas [41, 42]. Deep neural networks can also be combined [43, 44]. In addition, traditional gradient-based methods can be combined with metaheuristic algorithms. Inspired by these ideas, this paper proposes a hybrid MPA combined with the estimation of distribution algorithm for improving the basic MPA performance. And, we use Tent mapping and Gaussian random walk to further improve performance. The performance of HEGMPA was evaluated on the CEC2014 test suite and compared with five advanced algorithms. The superiority of the proposed algorithm is verified by numerical analysis, convergence analysis, stability analysis, and statistical analysis.

The left part of this paper is organized as follows: A review of the MPA is presented in Section 2.  Section 3 shows a detailed description of the proposed algorithm. In Section 4, the effectiveness of the proposed improvement strategy is verified using CEC2014 test suite. Finally, we summarize this work in Section 4 and present directions for future research.

## 2. Marine Predators Algorithm (MPA)

In this section, the procedure of the basic MPA is presented. MPA is a novel swarm-based metaheuristic algorithm mainly inspired by the different foraging strategies of marine predators and the optimal encounter rate strategy between the predator and prey.

Similar to most metaheuristics, the initial solution of MPA is distributed as evenly as possible in the search space. The initialization formula is as follows:

(1) $$\begin{array}{c} X\left(1\right)=X_{\min}+r_{1}\times \left(X_{\max}-X_{\min}\right), \end{array}$$

where *X*_min_ and *X*_max_ denote the upper and lower bounds of the population variables, respectively. *r*_1_ ∈ (0,1) is a random vector obeying a uniform distribution.

The MPA search process is divided into three phases based on different speed ratios: (1) a high-speed phase, where the prey speed is faster than the predator speed; (2) a unit speed ratio phase, where the prey speed and the predator speed are similar; and (3) a low-speed phase, where the prey speed is slower than the predator speed. In each stage, the movement of the predator and prey in nature is imitated separately.

**Phase 1 .**

The high-speed phase occurs at the beginning of the iteration, when the prey obeys Brownian motion and the predator performs mainly exploratory behaviour. The mathematical model for this phase is shown as follows:

(2) $$\begin{array}{c} \text{while iter}<\frac{1}{3}{\text{iter}}_{\max}, \end{array}$$

(3) $$\begin{array}{c} \overset{⟶}{Prey_{i}}=\overset{⟶}{Prey_{i}}+P\cdot \overset{⟶}{R}⊗\overset{⟶}{stepsize_{i}}, \end{array}$$

where $\overset{⟶}{R_{B}}$ is a random vector that follows a normal distribution based on Brownian motion. *P* is a constant taking the value 5. $\overset{⟶}{R}\in \left(\text{0,}1\right)$ is a uniformly distributed random vector. iter denotes the number of current iterations. iter_max_ denotes the maximum number of iterations.

**Phase 2 .**

In the second stage, both exploitation and exploration are required, so the stock is divided into two parts. One part is used for exploitation, and the other for exploration. The prey is used for the exploitation process and the predator for the exploration process. The mathematical model of this stage is described as follows:

(4) $$\begin{array}{c} \text{while}\frac{1}{3}{\text{iter}}_{\max}\leq \text{iter}<\frac{2}{3}{\text{iter}}_{\max}. \end{array}$$

The first part of the population carries out exploitation behaviour.

(5) $$\begin{array}{c} \overset{⟶}{stepsize_{i}}=\overset{⟶}{R_{L}}⊗\left(\overset{⟶}{Elite_{i}}-\overset{⟶}{R_{L}}⊗\overset{⟶}{Prey_{i}}\right)\text{,}\;i=1\text{,}\ldots \text{,}n, \end{array}$$

(6) $$\begin{array}{c} \overset{⟶}{Prey_{i}}=\overset{⟶}{Prey_{i}}+P\cdot \overset{⟶}{R}⊗\overset{⟶}{stepsize_{i}}, \end{array}$$

where $\overset{⟶}{R_{L}}$ is a random vector obeying a Lévy distribution.

The second part of the population performs exploratory behaviour.

(7) $$\begin{array}{c} \overset{⟶}{stepsize_{i}}=\overset{⟶}{R_{B}}⊗\left(\overset{⟶}{R_{B}}⊗\overset{⟶}{Elite_{i}}-\overset{⟶}{Prey_{i}}\right)\text{,}\;i=1\text{,}\ldots \text{,}n, \end{array}$$

(8) $$\begin{array}{c} \overset{⟶}{Prey_{i}}=\overset{⟶}{Elite_{i}}+P\cdot \text{CF}⊗\overset{⟶}{stepsize_{i}}, \end{array}$$

where CF=(1 − (iter/iter_max_))^2(iter/iter_max_)^ is an adaptive parameter that controls the predator step size.

**Phase 3 .**

As the last phase, the exploitation process is mainly carried out and the mathematical model of this phase is described as follows:

(9) $$\begin{array}{c} \text{while iter}>\frac{2}{3}{\text{iter}}_{\max}, \end{array}$$

(10) $$\begin{array}{c} \overset{⟶}{Prey_{i}}=\overset{⟶}{Elite_{i}}+P\cdot \text{CF}⊗\overset{⟶}{stepsize_{i}}. \end{array}$$

**Figure 1:**
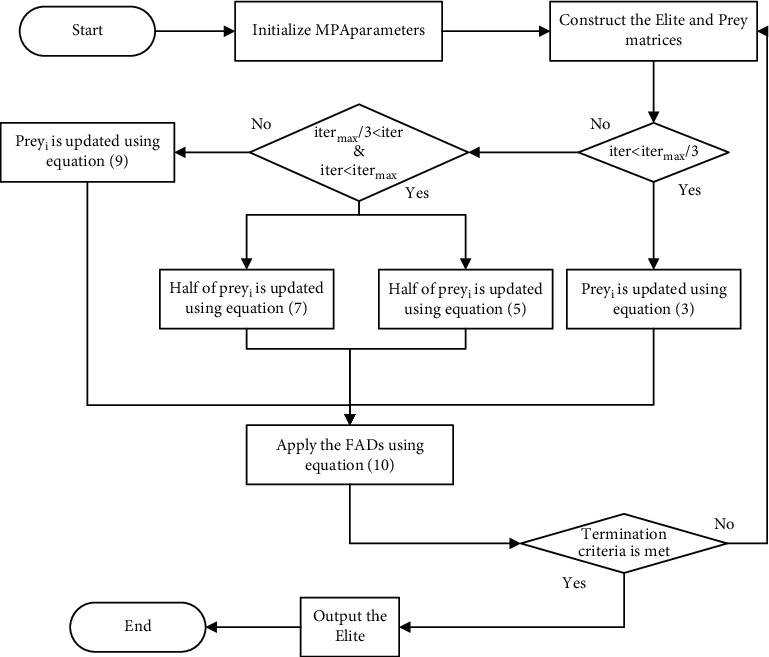
Flow chart of MPA.

In addition, environmental issues can cause changes in predator behaviour, and fish aggregating devices (FADs) are a factor that affects predator behaviour and are seen as a local optimum in this search area, assuming that the local optimum can be jumped out of by longer steps. The effect of FADs can be expressed mathematically as follows:

(11) $$\begin{array}{c} \overset{⟶}{Prey_{i}}=\left\{\begin{array}{cc} \overset{⟶}{Prey_{i}}+\text{CF}\left[\overset{⟶}{X_{\min}}+\overset{⟶}{R}⊗\left(\overset{⟶}{X_{\max}}-\overset{⟶}{X_{\min}}\right)\right]⊗\overset{⟶}{U}, & \text{if }r\leq \text{FADs},\\ \overset{⟶}{Prey_{i}}+\left[\text{FADs}\left(1-r\right)+r\right]\left(\overset{⟶}{Prey_{r1}}-\overset{⟶}{Prey_{r2}}\right)\text{,} & \text{if }r>\text{FADs}, \end{array}\right. \end{array}$$

where FADs=0.2 denotes the probability that FADs affect the optimization process. $\overset{⟶}{U}$ is a binary vector including 0 or 1. When a random vector from 0 to 1 is generated and is less than 0.2, the array is changed to 0, and vice versa. *r* ∈ (0,1) is a uniformly distributed random number. $\overset{⟶}{Prey_{r1}}$ and $\overset{⟶}{Prey_{r2}}$ are two randomly selected individuals. The pseudo-code for the MPA is shown in Algorithm 1 and Figure 1.

## 3. The Proposed MPA Variant

The basic MPA uses only the best individuals for iterative search, not making full use of valid information from the remaining individuals, resulting in reduced population diversity. The FADs process is performed at each iteration, which increases the computational cost. We use three strategies to improve the performance of the algorithm. Firstly, we take advantage of the good traversal and randomness of chaotic mapping to generate the initial solution of the population and increase the population diversity. Secondly, we use EDA to sample the dominant population information and correct the evolutionary direction. A Gaussian wandering strategy is used to enhance the population diversity when the algorithm stalls, helping the algorithm to jump out of the local optimum. Finally, a greedy strategy is used to ensure that the algorithm converges efficiently.

### 3.1. Population Initialization Based on Cubic Mapping

The initial population of most current intelligent optimization algorithms is randomly generated in the search space, and the quality of the initialized population has a great impact on the efficiency of the optimization algorithm. A uniformly distributed population is conducive to expanding the search range and thus improving the convergence speed and accuracy of the algorithm.

**Figure 2:**
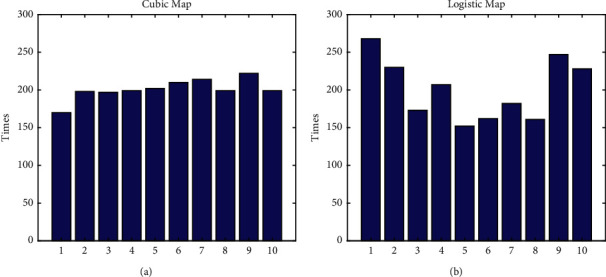
Mapping contrast diagram. (a) Logistic mapping values. (b) Cubic mapping values.

MPA, like other intelligent algorithms, suffers from a reduction in population diversity late in the iteration when solving complex problems, which can easily fall into local optima leading to premature maturity resulting in poor convergence accuracy. To improve its global search capability and avoid the reduction of population diversity in the postiteration period, the chaos operator is used to initialize the population, considering that it has the characteristics of randomness and regularity and can traverse all states within a certain range without repetition, so the cubic mapping chaos operator is used.  Figure 2 shows the effect of cubic mapping and logistic mapping.

The cubic mapping formula is shown as follows:

(12) $$\begin{array}{c} y\left(n+1\right)=4y{\left(n\right)}^{3}-3y\left(n\right),\;-1<y\left(n\right)<1,y\left(n\right)\neq 0,n=0,1,2,\ldots . \end{array}$$

The cubic mapping is used to initialize the prey population by generating a random vector of −1 to 1 in each dimension as the first individual, then iterating over each dimension of the first individual to obtain the remaining *M* − 1 individuals using equation (12), and finally mapping the values of the variables generated by the cubic mapping onto the prey individuals using

(13) $$\begin{array}{c} {\text{Prey}}_{i,j}=X_{\min}+\left(X_{\max}-X_{\min}\right)\times \frac{\left(y_{ij}+1\right)}{2}. \end{array}$$

### 3.2. Estimation of Distribution Algorithm

The estimation of distribution algorithm is an algorithm that uses probability models to represent relationships between individuals. EDA has been employed for hybridization with other algorithms and has achieved better results [45, 46]. It uses the current dominant population to calculate a probability distribution model and generates a new population of children based on the probability distribution model sampling, thus iterating continuously to eventually obtain the optimal solution. In this paper, the distribution model is estimated using a weighted maximum likelihood estimation method and the top one-half population with better performance is taken as the dominant population. The mathematical model of the algorithm is described as follows:

(14) $$\begin{array}{c} \overset{⟶}{Prey_{\text{mean}}}=\sum_{i=1}^{\text{NP}/2}\omega_{i}\times \overset{⟶}{Prey_{i}}, \end{array}$$

(15) $$\begin{array}{c} \omega_{i}=\frac{\ln\left(\text{NP}/2+0.5\right)-\ln\left(i\right)}{\sum_{i=1}^{\text{NP}/2}\left(\ln\left(\text{NP}/2+0.5\right)-\ln\left(i\right)\right)}, \end{array}$$

(16) $$\begin{array}{c} Cov\left(i\right)=\frac{1}{\text{NP}/2}\sum_{i=1}^{\text{NP}/2}\left(\overset{⟶}{Prey_{i}}-\overset{⟶}{Prey_{\text{mean}}}\right)\times {\left(\overset{⟶}{Prey_{i}}-\overset{⟶}{Prey_{\text{mean}}}\right)}^{T}, \end{array}$$

(17) $$\begin{array}{c} \overset{⟶}{Prey_{i}}=\text{Gaussian}\left(\overset{⟶}{Prey_{\text{mean}}}\text{,}Cov\left(i\right)\right)+\text{rand}\times \left(\overset{⟶}{Prey_{\text{mean}}}-\overset{⟶}{Prey_{i}}\right)\text{,} \end{array}$$

where $\overset{⟶}{Prey_{\text{mean}}}$ denotes the weighted mean of the dominant population. NP is the population size. *ω*_*i*_ denotes the weight coefficients in the dominant population in the descending order of fitness values. **Cov** is the weighted covariance matrix of the dominant population.

### 3.3. Medium Solution Gaussian Random Walk

A random walk strategy is used to help the algorithm jump out of stagnation and enhance its exploration capabilities when it falls into a local optimum late in the iteration. A stalled algorithm is considered to have stalled if the average fitness of the top half of the dominant population does not change in two consecutive iterations, and the random walk strategy is then used to update the population. The random walk strategy is a probabilistic model that simulates the random movement of organisms in nature and is widely used in the design and improvement of various optimization algorithms. In this paper, we propose a Gaussian random walk strategy for constructing new offspring using a medium population.

As the vector of differences between dominant and intermediate populations can improve the diversity of populations, information on intermediate populations is considered in this paper. The sampling points are related to the relative positions of the medium and dominant populations, extending the search range and providing a strong exploration capability. The mathematical model of the strategy is described as follows:

(18) $$\begin{array}{c} \overset{⟶}{Prey_{i}}=\text{Gaussian}\left(\overset{⟶}{Prey_{i}},\sigma \right)+\text{rand}\times \overset{⟶}{Prey_{r_{4}}^{\text{best}}}-\text{rand}\times \overset{⟶}{Prey_{r_{3}}^{\text{medium}}},\\ \sigma =\left|\overset{⟶}{Prey_{r_{4}}^{\text{best}}}-\overset{⟶}{Prey_{r_{3}}^{\text{medium}}}\right|, \end{array}$$

where $\overset{⟶}{Prey^{\text{best}}}$ and $\overset{⟶}{Prey^{\text{medium}}}$ represent randomly selected individuals from the dominant and intermediate populations, respectively.

At the end of each iteration, HEGMPA used a greedy strategy to retain the best NP individuals in the parent and offspring, thus forming a new population, which facilitated the global convergence ability of HEGMPA. In summary, the flow chart of the improved algorithm proposed in this paper is shown in the following. The pseudo-code for the HEGMPA is shown in Algorithm 2.

## 4. Simulation Experiments and Analysis of Results

To comprehensively validate the performance of the improved algorithms, we first verify the effectiveness of different improvement strategies and then verify the superiority and competitiveness of the improved algorithms by comparing them with recently proposed ones.

The CEC2014 test suite contains 30 test functions, which can be divided into four categories of test functions according to different characteristics: F1–F3 for single-peaked functions, F4–F16 for multipeaked functions, F17–F22 for mixed functions, and F23–F30 for combined functions. The definitions and optimal values of the functions are shown in Table 1. In the CEC2014 test, the maximum number of iterations is 600 and the population size is 500. All algorithms were run independently 51 times to record statistical values. The program was run on a MATLAB 2016b platform.

### 4.1. Comparison of HEGMPA Improvement Strategies

HEGMPA was compared with MPA-1 using cubic mapping to initialize the population, MPA-2 with fused EDA, MPA-3 using a moderately solved Gaussian random wandering strategy, and basic MPA to verify the effectiveness of the different improvement strategies.  Table 2 records the mean error, standard deviation, and ranking of the different algorithms for solving the test functions. The last column shows the average ranking of each algorithm.

From Table 2, HEGMPA with a full search strategy has the best search performance, while the basic MPA ranks last. Specifically, for unimodal test functions F1–F3, MPA-2, which only incorporates the EDA algorithm, performs similarly to HEGMPA in unimodal functions and far outperforms the other compared algorithms, indicating that incorporating the EDA strategy can effectively improve the development capability of the algorithm. For the multimodal functions F4–F16, HEGMPA and EDA-2 also ranked in the top two positions, while MPA was the least effective, suggesting that using dominant population information to generate offspring is beneficial in enhancing the diversity of individuals in the population. For the combinatorial functions F17–F30, HEGMPA only underperformed MPA-2 on F21, F24, and F26, indicating that the Tent chaotic mapping and moderately solved Gaussian random walk strategies can improve the algorithm's performance in solving complex combinatorial functions and effectively help the algorithm to jump out of the local optimum when the algorithm stalls. In summary, the improvement strategy proposed in this paper can effectively improve the MPA optimality finding performance.

### 4.2. An Analysis of HEGMPA Compared with Other Algorithms

To further illustrate the superiority of the improved algorithms, five algorithms, TLMPA, VCS [47], MMPA [48], CPIJADE [49], and HFPSO [50], are selected for comparison with HEGMPA. CPIJADE is an improved JADE algorithm using a new framework. HFPSO is an improved particle swarm algorithm mixed with the firefly algorithm. To ensure fairness, the parameters of each algorithm are referred to the original literature, as shown in Table 3. In the experiments, NP = 500, dim = 30, and the maximum number of evaluations is 300,000.  Table 4 records the average error and ranking of each algorithm in each test function for 51 independent runs.

The analysis in Table 4 shows that for single-peaked test functions F1–F3, HEGMPA outperforms all the comparative algorithms and can consistently find the optimal values of these three test functions, demonstrating the advantage of HEGMPA in solving pathological functions and once again verifying that the improved strategy can effectively improve the development capability of the algorithm. For the multipeaked test functions F4–F16, HEGMPA also outperforms all the comparative algorithms, indicating that the improved algorithms can maintain good enough population diversity to avoid falling into local optima. For complex combinatorial functions, each algorithm has its own advantages and disadvantages. HEGMPA obtains optimal solutions on F17–F22, F26, and F29–F30. VCS achieves better solutions on F23–F25. MMPA outperforms the other comparative algorithms on F23, F25, and F27–F28. CPIJADE performs best on F22. F17–F30 are more complex combinatorial test functions, and HEGMPA achieves the best results on eight of them, providing better evidence of HEGMPA's potential to solve complex optimization problems in the real world.

**Figure 3:**
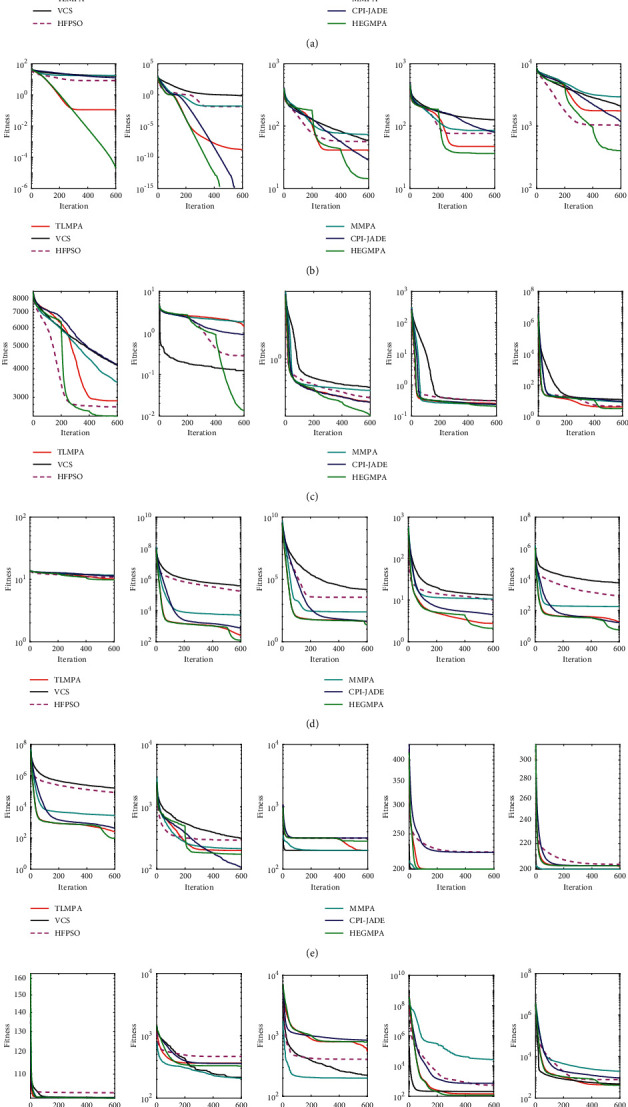
Convergence curves of CEC2014. (a) F1–F5. (b) F6–F10. (c) F11–F15. (d) F16–F20. (e) F21–F25. (f) F26–F30.

To further illustrate the convergence performance of the algorithms, Figure 3 shows the average error convergence curves of the six algorithms for solving the CEC2014 test set. HEGMPA has better convergence accuracy and faster convergence on F1–F4, F6–F7, F13, and F16–F21. In solving F5, F8–F10, and F12, HEGMPA converges faster and with better convergence accuracy in the later part of the iteration, although the convergence speed is slower in the early part of the iteration. In summary, HEGMPA outperforms the comparison algorithms in terms of convergence accuracy and convergence speed.

**Figure 4:**
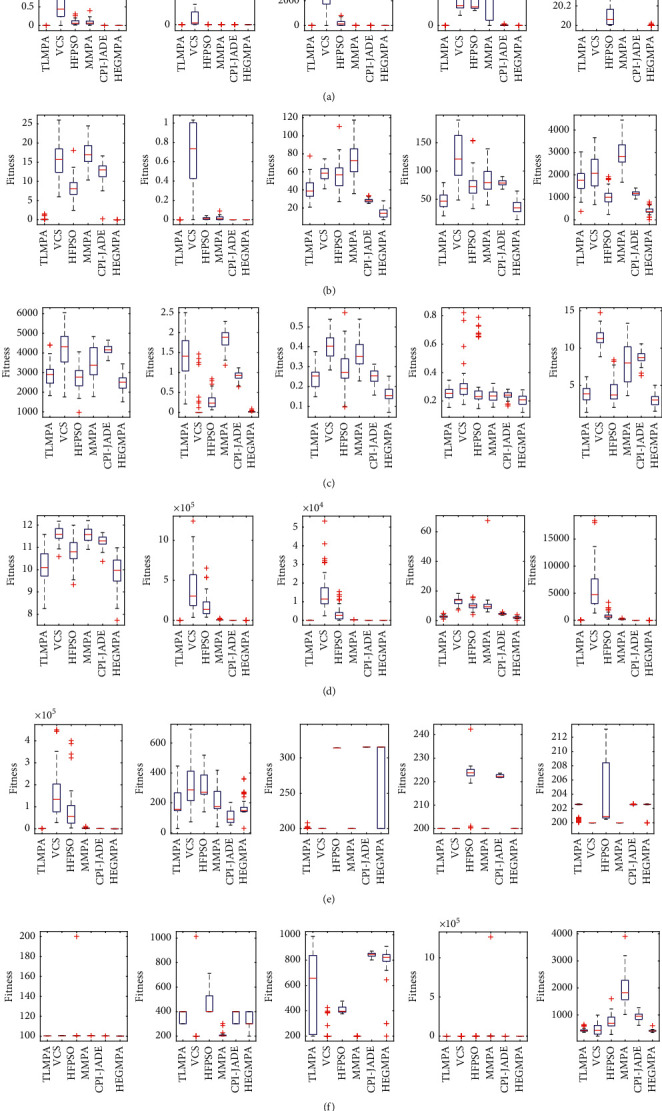
Box diagram of CEC2014. (a) F1–F5. (b) F6–F10. (c) F11–F15. (d) F16–F20. (e) F21–F25. (f) F26–F30.

To analyse the distributional properties of the solutions solved by the improved algorithms, box plots were drawn based on the results of 51 independent solutions for each algorithm, as shown in Figure 4. For each algorithm, the centre marker of each box indicates the median of the results of 51 solved functions, the bottom and top edges of the box indicate first- and third-degree points, and the symbol “+” indicates bad values that are not inside the box. As can be seen from Figure 4, HEGMPA has no outliers when solving 17 of the test functions (F1–F3, F7–F9, F11, F13–F15, F18, F21, F23–F24, F26–F27, and F29), indicating that the distribution solved by HEGMPA is very concentrated; meanwhile, for the other test functions where bad values exist, the HEGMPA has a small median, indicating that the quality of HEGMPA's solutions is relatively better. Therefore, the improved algorithm proposed in this paper has a strong robustness.

To avoid chance in testing, this paper uses the Wilcoxon signed-rank test to verify whether the improved algorithms are statistically significantly different from the comparison algorithms.  Table 5 presents the results of the Wilcoxon signed-rank sum test for each algorithm and HEGMPA. In the table, “+” indicates that HEGMPA outperforms the comparison algorithm in terms of optimization results, “−” indicates poorer results, “=” indicates similar results, and the symbol “*R*+” is a positive rank value indicating the extent to which HEGMPA is better than the comparison algorithm and “*R*−” indicates the opposite result. As can be seen in Table 4, HEGMPA outperformed the basic MPA in at least 23 of the 30 tested functions due to all comparison algorithms and in all tested functions, which statistically validates the excellent performance of the improved algorithm.

The computational efficiency of the algorithm is also another important aspect in evaluating the performance of the algorithm.  Table 6 lists the average time taken by each algorithm in solving the test function, and the last column lists the average ranking of each algorithm. We can learn that HEGMPA takes more time to compute, ranking only fourth, while MPA ranks third, due to the increased computational cost caused by the introduction of EDA, and the computation of the covariance matrix based on the Gaussian distribution model increases the computational time taken by the improved algorithm. Although the introduction of the improved strategy leads to an increase in the computational time consumed by the basic MPA, the performance improvement it brings is significant and therefore the computational time consumed by the HEGMPA proposed in this paper is acceptable.

## 5. Conclusions

In this paper, we propose a variant of MPA, called HEGMPA. The performance of the algorithm is improved using Tent mapping, distribution estimation strategy, and Gaussian random walk. To evaluate the effectiveness of the improved strategy and the superiority of HEGMPA, it was validated using the CEC2014 test suite. It was compared with five state-of-the-art algorithms through numerical analysis, convergence analysis, stability analysis, and statistical tests. The simulation results show that the HEGMPA algorithm balances development and exploration and is competitive with other algorithms. On the other hand, there is still room for improvement in HEGMPA. The effect of initialization of small populations needs to be investigated. The calculation of the covariance matrix increases the computational cost. Therefore, how to reduce the computational cost while maintaining performance is an issue that needs to be further investigated.

In future work, we plan to further apply HEGMPA to medical image recognition detection. In addition, we plan to develop a multiobjective version of HEGMPA to address optimization problems in other domains.

## Figures and Tables

**Algorithm 1:**
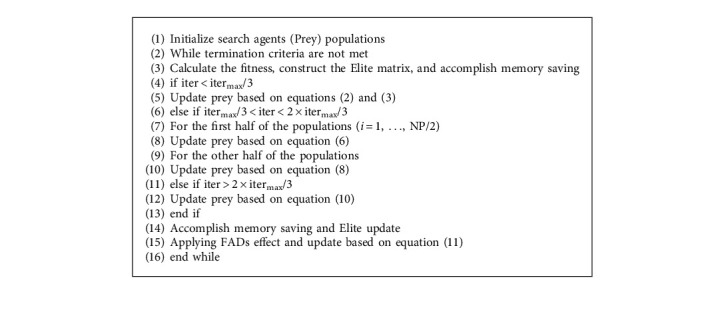
Pseudo-code of MPA.

**Algorithm 2:**
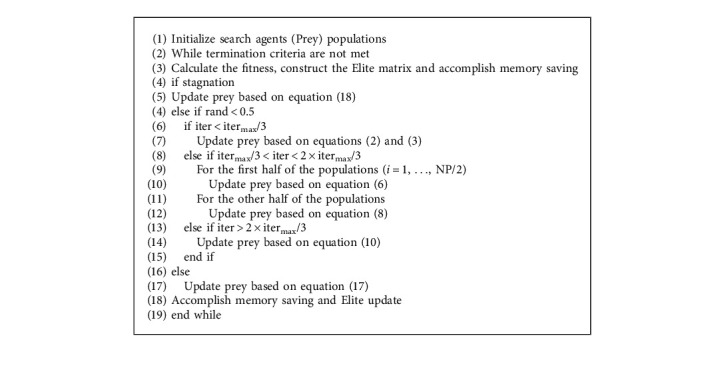
Pseudo-code of HEGMPA.

**Table 1:** Descriptions of CEC2014 test suite.

Type	No.	Function name	*F*_*i*_^*∗*^=*F*_*i*_(*x*^*∗*^)
Unimodal functions	1	Rotated high conditioned elliptic function	100
	2	Rotated bent cigar function	200
	3	Rotated discus function	300

Multimodal functions	4	Shifted and rotated Rosenbrock's function	400
	5	Shifted and rotated Ackley's function	500
	6	Shifted and rotated Weierstrass function	600
	7	Shifted and rotated Griewank's function	700
	8	Shifted Rastrigin's function	800
	9	Shifted and rotated Rastrigin's function	900
	10	Shifted Schwefel's function	1000
	11	Shifted and rotated Schwefel's function	1100
	12	Shifted and rotated Katsuura function	1200
	13	Shifted and rotated HappyCat function	1300
	14	Shifted and rotated HGBat function	1400
	15	Shifted and rotated expanded Griewank's plus Rosenbrock's function	1500
	16	Shifted and rotated expanded Schaffer's F6 function	1600

Hybrid functions	17	Hybrid function 1 (*N* = 3)	1700
	18	Hybrid function 2 (*N* = 3)	1800
	19	Hybrid function 3 (*N* = 4)	1900
	20	Hybrid function 4 (*N* = 4)	2000
	21	Hybrid function 5 (*N* = 5)	2100
	22	Hybrid function 6 (*N* = 5)	2200

Composite functions	23	Composition function 1 (*N* = 5)	2300
	24	Composition function 2 (*N* = 3)	2400
	25	Composition function 3 (*N* = 3)	2500
	26	Composition function 4 (*N* = 5)	2600
	27	Composition function 5 (*N* = 5)	2700
	28	Composition function 6 (*N* = 5)	2800
	29	Composition function 7 (*N* = 3)	2900
	30	Composition function 8 (*N* = 3)	3000
Search range: [−100,100]*D*			

**Table 2:** Statistics of the results of five algorithms in CEC2014 30D testing.

Function	MPA			MPA-1			MPA-2			MPA-3			HEGMPA		
	Mean	Std	Rank	Mean	Std	Rank	Mean	Std	Rank	Mean	Std	Rank	Mean	Std	Rank
F1	6.02*E* + 07	3.69*E* + 07	5	5.08*E* + 07	3.45*E* + 07	4	0.00*E* + 00	0.00*E* + 00	1	1.44*E* + 07	8.57*E* + 06	3	0.00*E* + 00	0.00*E* + 00	1
F2	3.86*E* + 09	3.19*E* + 09	5	3.67*E* + 09	2.61*E* + 09	4	0.00*E* + 00	0.00*E* + 00	1	9.32*E* + 07	5.60*E* + 08	3	0.00*E* + 00	0.00*E* + 00	1
F3	3.72*E* + 04	1.14*E* + 04	5	3.20*E* + 04	1.03*E* + 04	4	0.00*E* + 00	0.00*E* + 00	1	4.84*E* + 03	3.14*E* + 03	3	0.00*E* + 00	0.00*E* + 00	1

F4	4.10*E* + 02	1.63*E* + 02	4	4.24*E* + 02	1.61*E* + 02	5	2.01*E* − 14	9.15*E* − 14	1	1.56*E* + 02	3.80*E* + 01	3	6.02*E* − 14	1.94*E* − 13	2
F5	2.00*E* + 01	4.53*E* − 02	4	2.00*E* + 01	4.77*E* − 02	5	2.00*E* + 01	1.70*E* − 02	2	2.00*E* + 01	3.82*E* − 02	3	2.00*E* + 01	5.64*E* − 03	1
F6	2.58*E* + 01	2.53*E* + 00	5	2.50*E* + 01	2.65*E* + 00	4	2.79*E* − 05	9.58*E* − 05	1	1.57*E* + 01	2.23*E* + 00	3	3.00*E* − 02	2.10*E* − 01	2
F7	3.21*E* + 01	2.01*E* + 01	5	2.99*E* + 01	1.87*E* + 01	4	0.00*E* + 00	0.00*E* + 00	1	6.45*E* − 01	4.73*E* − 01	3	0.00*E* + 00	0.00*E* + 00	1
F8	1.27*E* + 02	2.53*E* + 01	5	1.22*E* + 02	2.78*E* + 01	4	1.38*E* + 01	5.45*E* + 00	2	1.52*E* + 01	7.02*E* + 00	3	1.35*E* + 01	5.94*E* + 00	1
F9	1.62*E* + 02	2.59*E* + 01	5	1.56*E* + 02	3.04*E* + 01	4	3.49*E* + 01	8.50*E* + 00	1	1.12*E* + 02	1.66*E* + 01	3	3.74*E* + 01	1.09*E* + 01	2
F10	2.09*E* + 03	6.43*E* + 02	5	1.83*E* + 03	4.49*E* + 02	4	3.53*E* + 02	1.80*E* + 02	2	2.99*E* + 02	1.82*E* + 02	1	3.79*E* + 02	1.87*E* + 02	3
F11	3.34*E* + 03	5.12*E* + 02	5	3.17*E* + 03	5.23*E* + 02	4	2.45*E* + 03	5.18*E* + 02	2	2.16*E* + 03	4.71*E* + 02	1	2.46*E* + 03	4.13*E* + 02	3
F12	1.76*E* − 01	7.58*E* − 02	4	1.84*E* − 01	9.38*E* − 02	5	1.13*E* − 02	7.21*E* − 03	1	6.78*E* − 02	3.21*E* − 02	3	1.36*E* − 02	1.04*E* − 02	2
F13	9.15*E* − 01	3.99*E* − 01	4	9.32*E* − 01	3.70*E* − 01	5	1.68*E* − 01	3.62*E* − 02	2	4.33*E* − 01	8.89*E* − 02	3	1.66*E* − 01	3.27*E* − 02	1
F14	5.12*E* + 00	6.14*E* + 00	5	4.23*E* + 00	5.98*E* + 00	4	2.18*E* − 01	3.74*E* − 02	2	2.64*E* − 01	7.52*E* − 02	3	2.05*E* − 01	4.06*E* − 02	1
F15	1.73*E* + 03	2.53*E* + 03	5	1.25*E* + 03	1.78*E* + 03	4	3.01*E* + 00	7.14*E* − 01	1	1.11*E* + 01	4.99*E* + 00	3	3.06*E* + 00	8.33*E* − 01	2
F16	1.17*E* + 01	5.54*E* − 01	4	1.18*E* + 01	4.79*E* − 01	5	9.98*E* + 00	5.98*E* − 01	2	1.05*E* + 01	4.45*E* − 01	3	9.75*E* + 00	5.74*E* − 01	1

F17	1.05*E* + 06	8.70*E* + 05	4	1.24*E* + 06	1.13*E* + 06	5	1.58*E* + 02	1.35*E* + 02	2	2.32*E* + 05	1.36*E* + 05	3	1.51*E* + 02	1.16*E* + 02	1
F18	6.31*E* + 03	6.69*E* + 03	5	5.34*E* + 03	4.39*E* + 03	4	2.20*E* + 01	7.42*E* + 00	2	1.20*E* + 03	4.22*E* + 02	3	2.18*E* + 01	6.38*E* + 00	1
F19	3.77*E* + 01	2.06*E* + 01	5	3.50*E* + 01	2.08*E* + 01	4	2.11*E* + 00	6.50*E* − 01	2	1.63*E* + 01	1.28*E* + 01	3	2.09*E* + 00	6.36*E* − 01	1
F20	1.09*E* + 04	6.82*E* + 03	5	1.07*E* + 04	6.90*E* + 03	4	6.96*E* + 00	2.48*E* + 00	2	4.58*E* + 03	3.85*E* + 03	3	6.79*E* + 00	2.45*E* + 00	1
F21	2.94*E* + 05	2.69*E* + 05	4	3.15*E* + 05	2.92*E* + 05	5	8.89*E* + 01	1.01*E* + 02	1	9.14*E* + 04	4.73*E* + 04	3	1.08*E* + 02	1.32*E* + 02	2
F22	4.90*E* + 02	1.89*E* + 02	4	5.08*E* + 02	2.00*E* + 02	5	1.99*E* + 02	9.35*E* + 01	2	3.76*E* + 02	1.40*E* + 02	3	1.58*E* + 02	3.03*E* + 01	1
F23	3.54*E* + 02	1.45*E* + 01	4	3.56*E* + 02	1.77*E* + 01	5	3.02*E* + 02	3.75*E* + 01	2	3.17*E* + 02	1.25*E* + 00	3	2.75*E* + 02	5.56*E* + 01	1

F24	2.70*E* + 02	9.95*E* + 00	5	2.57*E* + 02	9.11*E* + 00	4	2.00*E* + 02	2.26*E* − 07	1	2.00*E* + 02	4.02*E* − 05	3	2.00*E* + 02	2.56*E* − 07	2
F25	2.16*E* + 02	4.76*E* + 00	5	2.16*E* + 02	3.91*E* + 00	4	2.02*E* + 02	7.02*E* − 01	2	2.00*E* + 02	2.85*E* − 13	1	2.02*E* + 02	5.08*E* − 01	3
F26	1.01*E* + 02	2.88*E* − 01	2	1.03*E* + 02	1.41*E* + 01	3	1.00*E* + 02	2.98*E* − 02	1	1.06*E* + 02	2.37*E* + 01	4	1.14*E* + 02	3.47*E* + 01	5
F27	4.38*E* + 02	2.12*E* + 01	4	4.49*E* + 02	3.29*E* + 01	5	3.29*E* + 02	4.60*E* + 01	2	4.06*E* + 02	2.70*E* + 00	3	3.08*E* + 02	3.37*E* + 01	1
F28	1.32*E* + 03	2.27*E* + 02	5	1.30*E* + 03	2.15*E* + 02	4	7.82*E* + 02	1.45*E* + 02	2	1.13*E* + 03	3.09*E* + 02	3	7.37*E* + 02	1.70*E* + 02	1
F29	1.48*E* + 04	4.26*E* + 03	4	4.52*E* + 05	2.23*E* + 06	5	1.23*E* + 02	2.63*E* + 01	2	2.52*E* + 03	1.02*E* + 03	3	1.21*E* + 02	2.49*E* + 01	1
F30	2.99*E* + 04	2.69*E* + 04	4	3.12*E* + 04	2.62*E* + 04	5	4.14*E* + 02	2.97*E* + 01	2	6.65*E* + 03	2.77*E* + 03	3	4.12*E* + 02	4.33*E* + 01	1
Average rank	4.50			4.37			1.60			2.83			1.56		

**Table 3:** Algorithm parameter setting.

Algorithm	Parameter setting
TLMPA	*r*_3_ ∈ (0,1), *r*_4_ ∈ (0,1), *r*_5_ ∈ (0,1)
VCS	*λ*=0.5, *σ*=0.3
HFPSO	*c*_1_=*c*_2_=1.49445, *a*=0.2, *B*_0_=2
	*γ*=1, *w*_*i*_=0.9, *w*_*f*_=0.5
MMPA	*jr*=0.3, *w*=10
CPI-JADE	*Cr*=0.5, *F*=0.5, *c*=0.1, *p*=0.5

**Table 4:** Statistics of the results of six algorithms in CEC2014 30D testing.

Function	TLMPA		VCS		HFPSO		MMPA		CPIJADE		HEGMPA	
	Mean	Rank	Mean	Rank	Mean	Rank	Mean	Rank	Mean	Rank	Mean	Rank
F1	9.88*E* − 03	3	6.12*E* + 06	6	8.59*E* + 05	4	8.82*E* + 05	5	1.10*E* − 04	2	0.00*E* + 00	1
F2	3.18*E* − 04	3	4.90*E* + 05	6	1.76*E* + 03	5	4.96*E* + 02	4	1.57*E* − 09	2	0.00*E* + 00	1
F3	1.27*E* − 05	3	2.77*E* + 03	6	1.94*E* + 02	5	3.50*E* + 00	4	5.74*E* − 11	2	0.00*E* + 00	1

F4	5.62*E* − 04	2	4.61*E* + 01	4	4.83*E* + 01	5	5.78*E* + 01	6	6.21*E* − 01	3	1.56*E* − 14	1
F5	2.09*E* + 01	6	2.07*E* + 01	4	2.01*E* + 01	2	2.09*E* + 01	5	2.06*E* + 01	3	2.00*E* + 01	1
F6	1.09*E* − 01	2	1.59*E* + 01	5	8.20*E* + 00	3	1.73*E* + 01	6	1.24*E* + 01	4	1.98*E* − 05	1
F7	1.60*E* − 09	3	6.85*E* − 01	6	1.30*E* − 02	4	1.62*E* − 02	5	0.00*E* + 00	1	0.00*E* + 00	1
F8	4.11*E* + 01	3	5.81*E* + 01	5	5.64*E* + 01	4	7.35*E* + 01	6	2.83*E* + 01	2	1.43*E* + 01	1
F9	4.71*E* + 01	2	1.26*E* + 02	6	7.58*E* + 01	3	8.49*E* + 01	5	7.93*E* + 01	4	3.60*E* + 01	1
F10	1.75*E* + 03	4	2.09*E* + 03	5	1.04*E* + 03	2	2.93*E* + 03	6	1.17*E* + 03	3	4.03*E* + 02	1
F11	2.90*E* + 03	3	4.13*E* + 03	5	2.73*E* + 03	2	3.48*E* + 03	4	4.14*E* + 03	6	2.49*E* + 03	1
F12	1.42*E* + 00	5	1.23*E* − 01	2	2.81*E* − 01	3	1.82*E* + 00	6	9.10*E* − 01	4	1.36*E* − 02	1
F13	2.49*E* − 01	2	4.03*E* − 01	6	2.88*E* − 01	4	3.65*E* − 01	5	2.51*E* − 01	3	1.60*E* − 01	1
F14	2.52*E* − 01	4	3.07*E* − 01	6	3.07*E* − 01	5	2.35*E* − 01	2	2.39*E* − 01	3	2.05*E* − 01	1
F15	3.82*E* + 00	2	1.15*E* + 01	6	4.25*E* + 00	3	7.97*E* + 00	4	8.72*E* + 00	5	3.01*E* + 00	1
F16	1.01*E* + 01	2	1.16*E* + 01	5	1.08*E* + 01	3	1.16*E* + 01	6	1.13*E* + 01	4	9.84*E* + 00	1

F17	2.56*E* + 02	2	3.83*E* + 05	6	1.73*E* + 05	5	5.10*E* + 03	4	7.58*E* + 02	3	1.25*E* + 02	1
F18	3.95*E* + 01	2	1.50*E* + 04	6	3.67*E* + 03	5	2.44*E* + 02	4	4.25*E* + 01	3	2.26*E* + 01	1
F19	2.75*E* + 00	2	1.32*E* + 01	6	1.01*E* + 01	4	1.07*E* + 01	5	4.48*E* + 00	3	2.10*E* + 00	1
F20	1.80*E* + 01	3	5.96*E* + 03	6	8.32*E* + 02	5	1.78*E* + 02	4	1.67*E* + 01	2	5.90*E* + 00	1
F21	2.51*E* + 02	2	1.58*E* + 05	6	8.22*E* + 04	5	2.78*E* + 03	4	4.21*E* + 02	3	9.34*E* + 01	1
F22	1.99*E* + 02	3	3.14*E* + 02	6	2.91*E* + 02	5	2.13*E* + 02	4	1.07*E* + 02	1	1.74*E* + 02	2
F23	2.00*E* + 02	3	2.00*E* + 02	1	3.14*E* + 02	5	2.00*E* + 02	1	3.15*E* + 02	6	2.81*E* + 02	4
F24	2.00*E* + 02	4	2.00*E* + 02	1	2.23*E* + 02	6	2.00*E* + 02	2	2.22*E* + 02	5	2.00*E* + 02	3

F25	2.02*E* + 02	3	2.00*E* + 02	1	2.04*E* + 02	6	2.00*E* + 02	1	2.03*E* + 02	5	2.02*E* + 02	4
F26	1.00*E* + 02	2	1.00*E* + 02	5	1.02*E* + 02	6	1.00*E* + 02	4	1.00*E* + 02	3	1.00*E* + 02	1
F27	3.65*E* + 02	5	2.16*E* + 02	2	4.65*E* + 02	6	2.08*E* + 02	1	3.59*E* + 02	4	3.25*E* + 02	3
F28	5.51*E* + 02	4	2.21*E* + 02	2	4.09*E* + 02	3	2.00*E* + 02	1	8.44*E* + 02	6	7.87*E* + 02	5
F29	1.41*E* + 02	2	2.07*E* + 02	3	5.31*E* + 02	4	2.64*E* + 04	6	7.16*E* + 02	5	1.16*E* + 02	1
F30	4.34*E* + 02	2	4.72*E* + 02	3	7.67*E* + 02	4	1.95*E* + 03	6	9.30*E* + 02	5	4.15*E* + 02	1
Average rank	2.93		4.56		4.2		4.2		3.5		1.5	

**Table 5:** Wilcoxon signed-rank sum test results for CEC2014.

No.	MPA				TLMPA				VCS			
	*P* value	*R*+	*R*−	Win	*P* value	*R*+	*R*−	Win	*P* value	*R*+	*R*−	Win
F1	5.15*E* − 10	1326	0	+	5.15*E* − 10	1326	0	+	5.15*E* − 10	1326	0	+
F2	5.15*E* − 10	1326	0	+	5.15*E* − 10	1326	0	+	5.15*E* − 10	1326	0	+
F3	5.15*E* − 10	1326	0	+	5.15*E* − 10	1326	0	+	5.15*E* − 10	1326	0	+
F4	5.15*E* − 10	1326	0	+	5.15*E* − 10	1326	0	+	5.15*E* − 10	1326	0	+
F5	5.46*E* − 10	1325	1	+	5.15*E* − 10	1326	0	+	5.15*E* − 10	1326	0	+
F6	5.15*E* − 10	1326	0	+	5.15*E* − 10	1326	0	+	5.15*E* − 10	1326	0	+
F7	5.15*E* − 10	1326	0	+	5.15*E* − 10	1326	0	+	5.15*E* − 10	1326	0	+
F8	5.15*E* − 10	1326	0	+	5.15*E* − 10	1326	0	+	5.15*E* − 10	1326	0	+
F9	5.15*E* − 10	1326	0	+	1.00*E* − 04	1078	248	+	5.15*E* − 10	1326	0	+
F10	5.15*E* − 10	1326	0	+	5.46*E* − 10	1325	1	+	5.15*E* − 10	1326	0	+
F11	5.15*E* − 10	1326	0	+	8.47*E* − 04	1019	307	+	1.87*E* − 09	1304	22	+
F12	5.15*E* − 10	1326	0	+	5.15*E* − 10	1326	0	+	7.99*E* − 03	380	946	−
F13	5.15*E* − 10	1326	0	+	3.94*E* − 09	1291	35	+	5.15*E* − 10	1326	0	+
F14	5.15*E* − 10	1326	0	+	6.77*E* − 07	1193	133	+	4.17*E* − 09	1290	36	+
F15	5.15*E* − 10	1326	0	+	3.95*E* − 04	1041	285	+	5.15*E* − 10	1326	0	+
F16	5.15*E* − 10	1326	0	+	5.35*E* − 02	869	457	=	5.15*E* − 10	1326	0	+
F17	5.15*E* − 10	1326	0	+	3.45*E* − 03	975	351	+	5.15*E* − 10	1326	0	+
F18	5.15*E* − 10	1326	0	+	5.35*E* − 05	1094	232	+	5.15*E* − 10	1326	0	+
F19	5.15*E* − 10	1326	0	+	7.45*E* − 06	1141	185	+	5.15*E* − 10	1326	0	+
F20	5.15*E* − 10	1326	0	+	3.20*E* − 08	1253	73	+	5.15*E* − 10	1326	0	+
F21	5.15*E* − 10	1326	0	+	7.34*E* − 05	1086	240	+	5.15*E* − 10	1326	0	+
F22	5.15*E* − 10	1326	0	+	1.29*E* − 01	825	501	=	3.56*E* − 08	1251	75	+
F23	5.15*E* − 10	1326	0	+	3.58*E* − 07	120	1206	−	1.97*E* − 09	0	666	−
F24	5.15*E* − 10	1326	0	+	5.15*E* − 10	1326	0	+	5.15*E* − 10	0	1326	−
F25	5.15*E* − 10	1326	0	+	1.15*E* − 01	495	831	=	1.63*E* − 09	0	1176	−
F26	5.15*E* − 10	1326	0	+	1.14*E* − 08	1272	54	+	5.15*E* − 10	1326	0	+
F27	5.15*E* − 10	1326	0	+	1.32*E* − 06	1179	147	+	1.42*E* − 08	50	1225	−
F28	5.15*E* − 10	1326	0	+	7.94*E* − 05	242	1084	−	5.46*E* − 10	1	1325	−
F29	5.15*E* − 10	1326	0	+	7.94*E* − 05	1084	242	+	5.15*E* − 10	1326	0	+
F30	5.15*E* − 10	1326	0	+	1.29*E* − 01	825	501	=	1.74*E* − 01	808	518	=

+/−/=	30/0/0				24/2/4				23/6/1			
No.	HFPSO				MMPA				CPI-JAD*E*			
	P value	*R*+	R−	Win	P value	*R*+	R−	Win	P value	*R*+	R−	Win
F1	5.15*E* − 10	1326	0	+	5.15*E* − 10	1326	0	+	5.15*E* − 10	1326	0	+
F2	5.15*E* − 10	1326	0	+	5.15*E* − 10	1326	0	+	5.15*E* − 10	1326	0	+
F3	5.15*E* − 10	1326	0	+	5.15*E* − 10	1326	0	+	5.15*E* − 10	1326	0	+
F4	5.15*E* − 10	1326	0	+	5.15*E* − 10	1326	0	+	5.15*E* − 10	1326	0	+
F5	7.80*E* − 10	1319	7	+	5.15*E* − 10	1326	0	+	5.15*E* − 10	1326	0	+
F6	5.15*E* − 10	1326	0	+	5.15*E* − 10	1326	0	+	5.15*E* − 10	1326	0	+
F7	5.10*E* − 10	1326	0	+	5.15*E* − 10	1326	0	+	1.00*E*+00	0	0	=
F8	5.15*E* − 10	1326	0	+	5.15*E* − 10	1326	0	+	5.80*E* − 10	1324	2	+
F9	5.80*E* − 10	1324	2	+	5.80*E* − 10	1324	2	+	5.15*E* − 10	1326	0	+
F10	1.32*E* − 09	1310	16	+	5.15*E* − 10	1326	0	+	5.15*E* − 10	1326	0	+
F11	3.49*E* − 02	888	438	+	2.32*E* − 08	1259	67	+	5.15*E* − 10	1326	0	+
F12	5.15*E* − 10	1326	0	+	5.15*E* − 10	1326	0	+	5.15*E* − 10	1326	0	+
F13	1.18*E* − 09	1312	14	+	5.15*E* − 10	1326	0	+	8.77*E* − 10	1317	9	+
F14	3.82*E* − 04	1042	284	+	2.76*E* − 04	1051	275	+	7.78*E* − 06	1140	186	+
F15	6.24*E* − 06	1145	181	+	5.15*E* − 10	1326	0	+	5.15*E* − 10	1326	0	+
F16	2.41*E* − 07	1214	112	+	5.15*E* − 10	1326	0	+	5.15*E* − 10	1326	0	+
F17	5.15*E* − 10	1326	0	+	5.15*E* − 10	1326	0	+	5.15*E* − 10	1326	0	+
F18	5.15*E* − 10	1326	0	+	5.15*E* − 10	1326	0	+	5.80*E* − 10	1324	2	+
F19	5.15*E* − 10	1326	0	+	5.15*E* − 10	1326	0	+	5.15*E* − 10	1326	0	+
F20	5.15*E* − 10	1326	0	+	5.15*E* − 10	1326	0	+	5.15*E* − 10	1326	0	+
F21	5.15*E* − 10	1326	0	+	5.15*E* − 10	1326	0	+	1.25*E* − 09	1311	15	+
F22	4.94*E* − 09	1287	39	+	1.27*E* − 02	929	397	+	1.31*E* − 07	100	1226	−
F23	9.78*E* − 01	660	666	=	1.97*E* − 09	0	666	−	6.10*E* − 05	120	0	+
F24	5.15*E* − 10	1326	0	+	5.15*E* − 10	0	1326	−	5.15*E* − 10	1326	0	+
F25	7.50*E* − 01	629	697	=	1.63*E* − 09	0	1176	−	1.98*E* − 03	333	993	−
F26	1.25*E* − 09	1311	15	+	5.15*E* − 10	1326	0	+	6.53*E* − 10	1322	4	+
F27	5.80*E* − 10	1324	2	+	7.35*E* − 10	6	1320	−	1.92*E* − 05	1119	207	+
F28	7.35*E* − 10	6	1320	−	5.15*E* − 10	0	1326	−	2.06*E* − 04	1059	267	+
F29	5.15*E* − 10	1326	0	+	5.15*E* − 10	1326	0	+	5.15*E* − 10	1326	0	+
F30	1.67*E* − 09	1306	20	+	5.15*E* − 10	1326	0	+	5.15*E* − 10	1326	0	+
+/−/=	27/1/2				25/5/0				27/2/1			

**Table 6:** Time cost of seven algorithms in CEC2014 30D testing.

	MPA	TLMPA	VCS	HFPSO	MMPA	CPIJADE	HEGMPA
F1	2.80*E* + 00	2.19*E* + 00	2.52*E* + 00	1.32*E* + 00	2.71*E* + 01	3.51*E* + 00	3.24*E* + 00
F2	2.25*E* + 00	1.69*E* + 00	1.97*E* + 00	8.31*E*-01	2.58*E* + 01	3.09*E* + 00	2.63*E* + 00
F3	2.26*E* + 00	1.73*E* + 00	1.94*E* + 00	8.23*E*-01	2.65*E* + 01	3.15*E* + 00	2.68*E* + 00

F4	2.26*E* + 00	1.74*E* + 00	1.97*E* + 00	8.22*E*-01	2.75*E* + 01	3.18*E* + 00	2.61*E* + 00
F5	2.57*E* + 00	1.83*E* + 00	2.92*E* + 00	1.10*E* + 00	2.69*E* + 01	3.11*E* + 00	2.94*E* + 00
F6	2.69*E* + 01	2.78*E* + 01	3.25*E* + 01	2.52*E* + 01	6.61*E* + 01	2.83*E* + 01	2.74*E* + 01
F7	2.59*E* + 00	1.95*E* + 00	2.25*E* + 00	1.08*E* + 00	2.61*E* + 01	3.40*E* + 00	2.91*E* + 00
F8	2.12*E* + 00	1.49*E* + 00	1.80*E* + 00	6.31*E*-01	2.54*E* + 01	2.64*E* + 00	2.40*E* + 00
F9	2.54*E* + 00	1.99*E* + 00	2.17*E* + 00	1.06*E* + 00	2.73*E* + 01	3.12*E* + 00	2.87*E* + 00
F10	2.90*E* + 00	1.87*E* + 00	2.28*E* + 00	1.03*E* + 00	2.66*E* + 01	3.03*E* + 00	2.73*E* + 00
F11	3.28*E* + 00	2.35*E* + 00	3.18*E* + 00	1.58*E* + 00	2.79*E* + 01	3.52*E* + 00	3.15*E* + 00
F12	7.98*E* + 00	8.13*E* + 00	6.31*E* + 00	6.52*E* + 00	3.65*E* + 01	9.59*E* + 00	8.32*E* + 00
F13	2.28*E* + 00	1.54*E* + 00	1.93*E* + 00	8.16*E*-01	2.65*E* + 01	2.87*E* + 00	2.62*E* + 00
F14	2.26*E* + 00	1.43*E* + 00	1.91*E* + 00	7.92*E*-01	2.74*E* + 01	2.84*E* + 00	2.59*E* + 00
F15	2.60*E* + 00	2.05*E* + 00	2.25*E* + 00	1.11*E* + 00	2.62*E* + 01	3.22*E* + 00	2.99*E* + 00
F16	2.62*E* + 00	2.18*E* + 00	2.46*E* + 00	1.16*E* + 00	2.61*E* + 01	3.24*E* + 00	3.24*E* + 00

F17	3.02*E* + 00	1.99*E* + 00	2.57*E* + 00	1.25*E* + 00	2.63*E* + 01	3.50*E* + 00	3.17*E* + 00
F18	2.41*E* + 00	1.41*E* + 00	2.13*E* + 00	9.30*E*-01	2.58*E* + 01	3.14*E* + 00	2.85*E* + 00
F19	7.42*E* + 00	7.55*E* + 00	7.28*E* + 00	5.93*E* + 00	3.36*E* + 01	8.91*E* + 00	7.88*E* + 00
F20	2.47*E* + 00	1.56*E* + 00	2.44*E* + 00	1.02*E* + 00	2.61*E* + 01	3.17*E* + 00	2.83*E* + 00
F21	2.57*E* + 00	1.75*E* + 00	2.44*E* + 00	1.11*E* + 00	2.64*E* + 01	3.31*E* + 00	2.94*E* + 00
F22	3.19*E* + 00	2.78*E* + 00	3.15*E* + 00	1.67*E* + 00	2.70*E* + 01	4.06*E* + 00	3.55*E* + 00
F23	4.76*E* + 00	4.47*E* + 00	4.46*E* + 00	3.27*E* + 00	2.97*E* + 01	6.25*E* + 00	5.16*E* + 00

F24	4.60*E* + 00	3.32*E* + 00	3.29*E* + 00	2.11*E* + 00	2.78*E* + 01	4.51*E* + 00	6.73*E* + 00
F25	5.49*E* + 00	4.42*E* + 00	4.23*E* + 00	3.10*E* + 00	2.95*E* + 01	5.87*E* + 00	4.93*E* + 00
F26	3.10*E* + 01	3.09*E* + 01	4.76*E* + 01	2.86*E* + 01	7.00*E* + 01	3.17*E* + 01	3.08*E* + 01
F27	3.01*E* + 01	3.10*E* + 01	4.45*E* + 01	2.88*E* + 01	6.96*E* + 01	3.15*E* + 01	3.06*E* + 01
F28	5.96*E* + 00	6.00*E* + 00	6.32*E* + 00	4.39*E* + 00	3.09*E* + 01	7.00*E* + 00	6.34*E* + 00
F29	9.24*E* + 00	9.67*E* + 00	1.31*E* + 01	7.47*E* + 00	3.62*E* + 01	1.06*E* + 01	9.45*E* + 00
F30	4.68*E* + 00	4.66*E* + 00	5.09*E* + 00	3.18*E* + 00	2.94*E* + 01	5.76*E* + 00	5.06*E* + 00
Average rank	3.73	2.50	3.36	1.03	7.00	5.76	4.60

## Data Availability

The data used to support the findings of this study are included within the article.
